# A Tertiary Twist to the Transglutaminase Tale

**DOI:** 10.1371/journal.pbio.0050337

**Published:** 2007-12-27

**Authors:** Frits Koning

## Abstract

A novel structure shows tissue transglutaminase in its catalytically active form, revealing the mode of action of this crosslinking enzyme and facilitating the development of specific inhibitors for use in various diseases in which transglutaminases are implicated.

Tissue transglutaminase (TG2) is one of a family of enzymes that covalently crosslink proteins by binding a glutamine in one protein with a lysine in another [1]. There is strong evidence that links the deregulated expression of TG2 to a variety of diseases, and although the exact role of TG2 in neurodegenerative diseases is unclear [2], in celiac disease (CD), TG2 catalyses the formation of immunogenic peptides that play a crucial role in disease development [3,4]. Although x-ray crystal structures of transglutaminases have been reported previously, the catalytic site was hidden due to the protein's conformation. In this issue of *PLoS Biology*, a structure of TG2 is shown in which the enzyme is captured in its active state [5] with the catalytic site visible. This provides new insight into the mode of action of TG2 and offers novel opportunities for the development of TG2 inhibitors, which could have therapeutic applications. The paper also raises questions about what regulates TG2 activation and how TG2 activation influences disease development.

## The Role of TG2 in Celiac Disease

Patients with celiac disease are intolerant to gluten proteins in wheat and related cereals [3,4]. In healthy individuals, the small intestine is lined with villi, which create a larger surface for the uptake of nutrients from food. In approximately 1% of the population in the Western Hemisphere, gluten induces inflammatory immune responses in the small intestine that lead to the disappearance of these villi. Malabsorption, retarded growth, diarrhea, and stomach ache can result, and these are the characteristic symptoms associated with CD [3,4].

**Figure 1:**
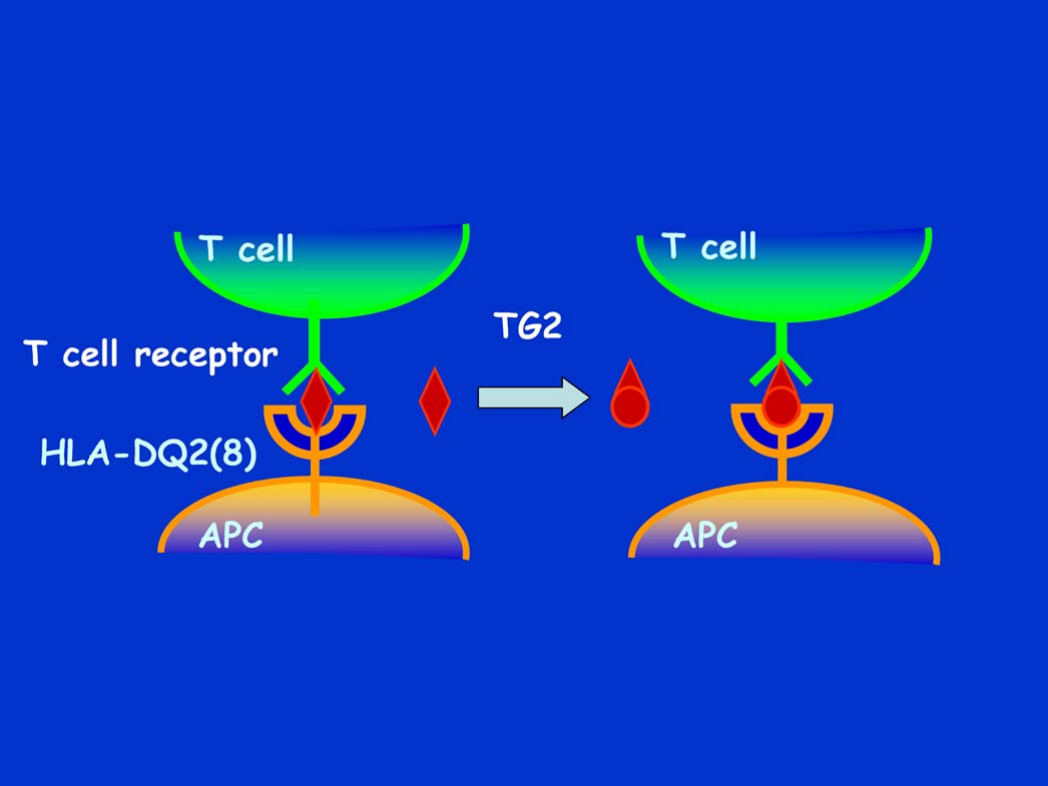
HLA-DQ2 and HLA-DQ8 Are Expressed on So-Called Antigen-Presenting Cells The function of antigen-presenting cells (APCs) is to bind peptides derived from pathogens and “present” these to the T cells of the immune system. T cells detect such HLA–peptide complexes through their T cell receptors. In celiac disease, T cells respond to gluten peptides bound to HLA-DQ2 or HLA-DQ8. Gluten-derived peptides (red diamond) bind only with low affinity to HLA-DQ2 and HLA-DQ8, but TG2 can modify such peptides, which turns them into high-affinity binders. Consequently, the HLA–gluten peptide complexes are more stable, which facilitates and enhances T cell responses to such peptides.

CD typically develops in human leukocyte antigen (HLA)-DQ2– and/or HLA-DQ8–positive individuals [3,4]. HLA molecules are receptors that specifically bind peptides and present these to the T cells of the immune system. When such peptides are derived from pathogens, an immune response is initiated to eradicate the pathogen. In the small intestine of patients with CD, erroneous T cell responses are made against gluten peptides bound to HLA-DQ2 and/or -DQ8 [6–9], as if they were pathogen derived, producing large amounts of interferon-gamma, which is a mediator of inflammation. However, it was previously unclear how HLA-DQ2 and HLA-DQ8 bind gluten peptides. These molecules prefer peptides containing one or more negatively charged amino acids, yet gluten proteins are essentially devoid of these amino acids [10–12]. This paradox was resolved when it was observed that TG2 converts the glutamine residues in gluten peptides into glutamic acid, introducing the negative charges required for binding to HLA-DQ2 or -DQ8 [13,14] (Figure 1). In fact, gluten and TG2 have a special relationship: due to the amino acid composition of glutens (~30% Q, ~17% P, rich in F and Y), the sequences QXPF and QXPY (X is any amino acid except P) are common and the Q is a target for TG2 [15]. TG2 modifies gluten at several positions, leading to the formation of a large number of gluten peptides that can bind to HLA-DQ2 and/or -DQ8 and induce T cell responses [15–17]. Together, these findings point to a crucial role of TG2 in the development of CD.

## Specific Contribution of Current Manuscript

Previous studies revealed that the GDP-bound form of TG2, which functions as a G-proteins within cells, is in a conformation where the active site is inaccessible and so the enzyme is catalytically inactive. In the current issue of *PLoS Biology*, Pinkas and colleagues report the structure of the catalytically active form of TG2 [5]. To achieve this, they have captured the enzyme in complex with an inhibitor based on a gluten-derived substrate for TG2, the pentapeptide PQLPF. By replacing the Q with an electrophilic trap (6-diazo-5-oxo-L-norleucine; don), a peptide was generated that covalently links itself to the cys residue in the active site of TG2, thus stabilizing it in its active state.

The novel structure reveals that the active state is fundamentally different from the inactive state. The complete C-terminal beta1- and beta2-barrels, which cover the N-terminal core domain in the catalytically inactive form, are in an extended conformation in the active form. This involves a 120-Å shift of the C-terminal residues, exposing the active site and the bound pentapeptide. This pentapeptide occupies one side of a hydrophobic tunnel that is bordered by two tryptophan residues and contains the catalytic cys residue, whereas the other side of the tunnel can accommodate the lysine residue, which will be covalently linked to the glutamine in the transamidation reaction. Several hydrogen bonds and hydrophobic interactions exist between TG2 and the pentapeptide PdonLPF. This is, in part, due to the trans conformation of the proline preceding the phenylalanine, which places the phenylalanine in a hydrophobic pocket. These interactions thus reveal why gluten peptides containing a QLPF or QLPY sequence are such good substrates for TG2.

Importantly, a similar large conformational shift is now expected to occur in other transglutaminases, suggesting a general mechanism involved in the activation of this family of enzymes.

## Perspective

As mentioned above, TG2 deamidates gluten peptides—a crucial step toward a full-blown T cell response against gluten [3,4]. Moreover, it is well established that TG2-specific autoantibodies are specific indicators of CD, because they are almost exclusively found in CD patients. It has been shown that TG2 can crosslink itself to gluten, and it has been speculated that if such complexes are taken up by B cells—which express surface immunoglobulin specific for TG2—the TG2 complexes would be internalized and degraded intracellularly. This would release gluten peptides that subsequently can be bound to HLA-DQ2 molecules and presented to gluten-specific T cells, which would result in B cell activation and antibody production. In this way, gluten-specific T cells would drive the production of TG2-specific antibodies [21]. In the current manuscript, Pinkas and colleagues [5] suggest an interesting alternative possibility: that the large conformational change associated with TG2 activation may expose regions of TG2 that are normally shielded and that this leads to antibody formation.

All of these ideas underscore the fact that TG2 activation is a critical step in disease development. But how is TG2 activated and which circumstances promote a broad gluten-specific T cell response? There is a large pool of extracellular TG2 in the intestine but no evidence that it is catalytically active [23]. Pinkas and colleagues [5] now speculate that in CD, this extracellular TG2 might be activated by ligands of the Toll-like receptor (TLR) receptors, which are pattern-recognition receptors that alert the immune system to the presence of pathogens. However, TLR triggering is not unique to CD; it is part of the normal immune response to pathogens. So, transient TG2 activation is thus probably a natural and common phenomenon, as might be expected of an enzyme known to be involved in wound and tissue repair [1]. Similarly, evidence has been presented that indicates that certain gluten peptides might activate the innate immune system [24–26], which could likewise contribute to TG2 activation. But it is not clear why this would only occur in CD patients and not in healthy individuals. Further, most HLA-DQ2– and -DQ8–positive individuals do not generate gluten-specific T cells or develop disease, despite the fact that they would be capable of generating deamidated gluten peptides if TG2 is active [3,4]. Thus, there must be something fundamentally different when an individual does develop CD: what is the master switch that determines disease development?

The key issue is that even though the majority of gluten-reactive T cells in the intestine are specific for deamidated gluten, TG2 in the intestine is normally inactive, so no deamidated gluten peptides are generated. Then what activates TG2 and thereby initiates the deamidation of gluten? The answer may lie in the gluten-specific T cell response itself: some gluten-specific T cells respond to native gluten peptides [7,8,14], and such T cell responses could induce a low level of inflammation and the release of catalytically active TG2 from the damaged tissue [14] (Figure 1). Once this occurs, TG2 will generate a host of immunogenic gluten peptides. This may be particularly important in the early stages of disease development, because it would allow rapid epitope spreading: the diversification of a limited response to a few (native) gluten peptides to a broad response involving a multitude of peptides [8]. Obviously, the development of such a T cell response takes weeks or even months, and early in that process, no symptoms will be apparent. The sustained exposure to gluten will generate a large gluten-specific T cell compartment in the intestine that will ultimately be dominated by T cells that are specific for the most immunogenic peptides, i.e., deamidated peptides with the best HLA-DQ–binding properties.

Ultimately, the question remains as to what drives the initial gluten-specific T cell response. Immune responses in the intestine are tightly regulated to avoid deleterious responses against harmless protein antigens. Infections in the intestine, however, are common, and these will force the mucosal immune system to take appropriate steps to eradicate the pathogens. This involves the polarization of the immune system toward inflammation. And this may provide a window in which inflammatory, gluten-specific T cell responses may escape from the tight regulation that suppresses such responses. Depending on the nature, duration, and/or persistence of the infections, such responses may become uncontrollable and lead to disease, whereas under more favorable conditions, they can be ultimately suppressed. It is likely that the outcome is influenced by genetics: a recent genome-wide association study has provided compelling evidence that a gene locus containing four genes, including *IL2* and *IL21*, contributes to the chance of developing CD [27]. Clearly, the potential involvement of these immune-related *IL2* and *IL21* genes is interesting, but because the risk associated is small [27], it will be a major undertaking to understand how such gene variants influence the chance of disease development.

Finally, the structure of TG2 in its active form may allow the design of potent inhibitors for TG2. Such blockers could potentially be used for the treatment of diseases in which TG2 is thought to play a role, including neurodegenerative diseases and, of course, CD. Yet in CD, it is unclear if we would want to use them for this purpose, because a gluten-free diet is an effective and entirely safe treatment for CD. Given the overall poor understanding of the multiple functions of TG2 and its role in wound repair, there might be unwanted side effects that limit the use of such blockers for treatment of CD. Conversely, for the treatment of neurodegenerative diseases, where the role of TG2 is less clear, the severity of the diseases may warrant the use of such inhibitors, despite potential side effects.

In conclusion, the tertiary twist associated with TG2 activation opens doors that improve our understanding of the role of TG2 in disease development and may aid in the development of novel therapeutic options.

## Abbreviations

CD: celiac disease
HLA: human leukocyte antigen
TG2: tissue transglutaminase
TLR: Toll-like receptor
